# Supplementary material for: Mid-Arm Muscle Circumference or Body Weight-Standardized Hand Grip Strength in the GLIM Superiorly Predicts Survival in Chinese Colorectal Cancer Patients
Source: Nutrients. 2022 Dec 5;14(23):5166. doi: 10.3390/nu14235166 (PMC9739446; doi:10.3390/nu14235166)
Supplement: Supplementary file 1 [file nutrients-14-05166-s001.zip › nutrients-1999589-supplementary.pdf]

nutrients MDPI

Supplementary Materials:

Table S1. Inclusion and Exclusion Criteria for the INSCOC Projecta

Criteria type	Description
Inclusion	1) Age at least 18 years;<br>2) With length of hospital stay longer than 48 hours;<br>3) Diagnosed with one of the following 18 types of locally or metastatic malignant tumors: lung cancer, gastric cancer, liver cancer, colorectal cancer, breast cancer, esophageal cancer, cervical cancer, endometrial cancer, nasopharyngeal carcinoma, malignant lymphoma, leukemia, pancreatic cancer, ovarian cancer, prostate cancer, bladder cancer, brain tumors, biliary tract malignant tumors and gastrointestinal stromal tumors
Exclusion	1) With organ transplantation;<br>2) Pregnant woman;<br>3) Diagnosed with HIV infection or AIDS;<br>4) Admitted to the ICU at the beginning of recruitment.<br>5) If patients were hospitalized more than two times during investigation, only the data from the first survey were included

INSCOC, Investigation on Nutrition Status and its Clinical Outcome of Common Cancers (chictr.org.cn: ChiCTR1800020329).

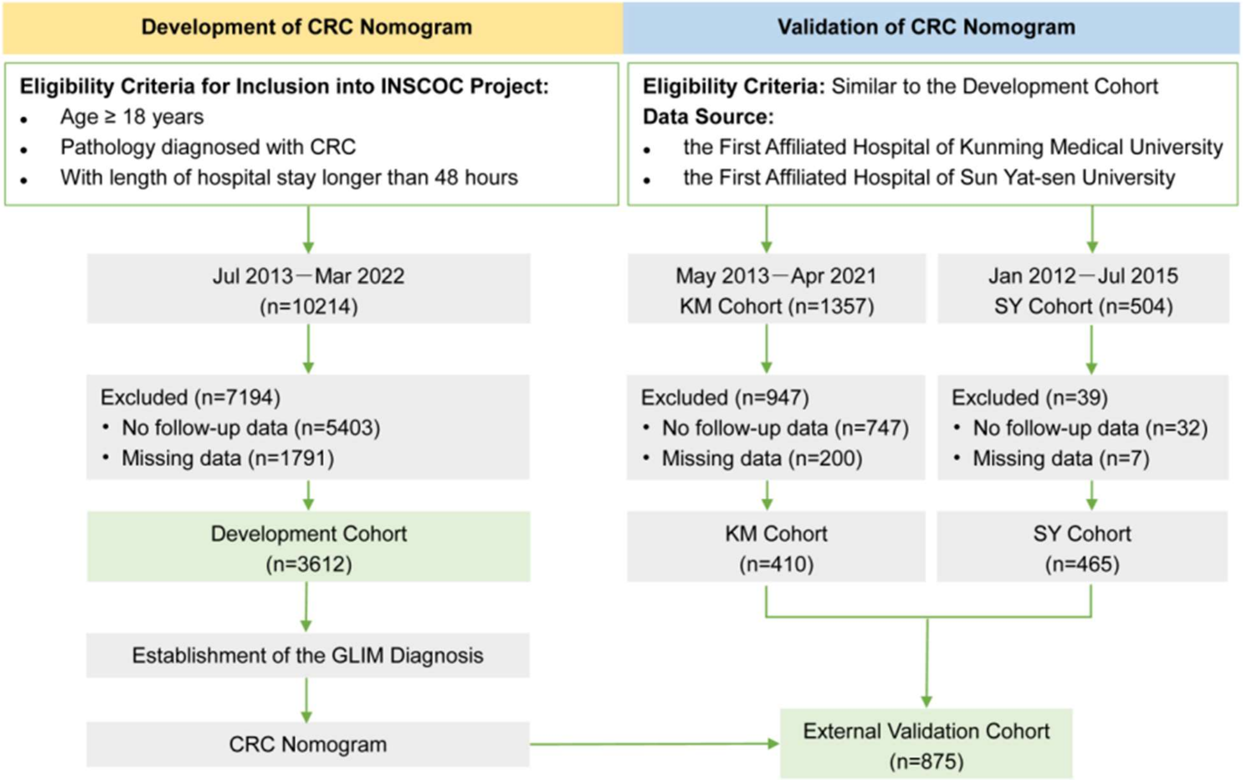

Figure S1. Overview and design of the study. CRC, colorectal cancer; INSCOC, the Investigation on Nutrition Status and its Clinical Outcome of Common Cancers; GLIM, the Global Leadership Initiative on Malnutrition.

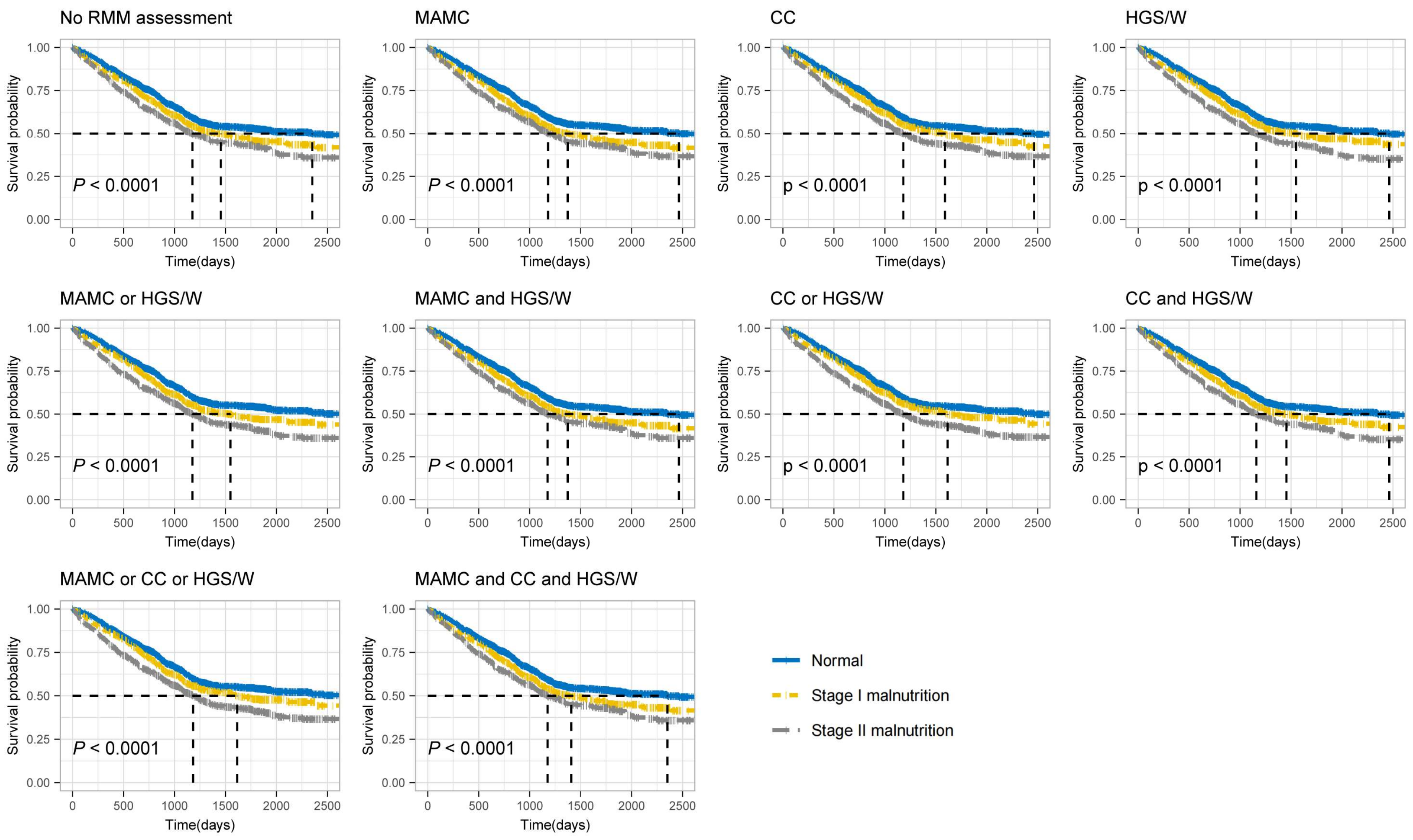

Figure S2. Kaplan-Meier curve analyses on the association between GLIM diagnosed malnutrition based on different RMM assessment and survival. GLIM, Global Leadership Initiative on Malnutrition; RMM, reduced muscle mass; MAMC, midarm muscle circumference; CC, calf circumference; HGS/W, hand grip strength/weight; *For no RMM assessment, only weight loss and body mass index were evaluated as phenotypic criteria for establishing the GLIM diagnosis.

Table S2. Detailed Baseline Characteristics of the development and validation cohort.

Characteristics	Development cohort			P	Validation cohort			P
	Normal (n=1892)	Moderate malnutrition (n=947)	Severe malnutrition (n=773)		Normal (n=402)	Moderate malnutrition (n=242)	Severe malnutrition (n=231)	
General information								
Age, years, mean±SD	63.12±11.95	64.80±12.63	65.58±13.20	<0.001	62.35±12.22	65.73±11.64	66.73±14.43	<0.001
Sex, male, n (%)	1125 (59.5)	564 (59.6)	484 (62.6)	0.291	234 (58.2)	147 (60.7)	134 (58.0)	0.781
Smoking, yes, n (%)	684 (36.2)	364 (38.4)	313 (40.5)	0.095	143 (35.6)	111 (45.9)	94 (40.7)	0.033
Alcohol drinker, yes, n (%)	348 (18.4)	181 (19.1)	151 (19.5)	0.765	42 (10.4)	37 (15.3)	31 (13.4)	0.180
Tea consumption, yes, n (%)	448 (23.7)	214 (22.6)	161 (20.8)	0.278	137 (34.1)	89 (36.8)	88 (38.1)	0.564
Residence, n (%)				0.215				0.925
Capital city	584 (30.9)	295 (31.2)	242 (31.3)		107 (26.6)	68 (28.1)	72 (31.2)	
Prefecture level cities	399 (21.1)	184 (19.4)	168 (21.7)		112 (27.9)	68 (28.1)	63 (27.3)	
County-level city	562 (29.7)	280 (29.6)	196 (25.4)		70 (17.4)	39 (16.1)	39 (16.9)	
Rural areas	347 (18.3)	188 (19.9)	167 (21.6)		113 (28.1)	67 (27.7)	57 (24.7)	
Education, n (%)				<0.001				0.197
Primary school and below	439 (23.2)	303 (32.0)	227 (29.4)		97 (24.1)	76 (31.4)	69 (29.9)	
Junior middle school	559 (29.5)	254 (26.8)	217 (28.1)		165 (41.0)	91 (37.6)	88 (38.1)	
Senior high school	530 (28.0)	222 (23.4)	198 (25.6)		75 (18.7)	50 (20.7)	46 (19.9)	
University degree or above	364 (19.2)	168 (17.7)	131 (16.9)		65 (16.2)	25 (10.3)	28 (12.1)	
Occupation, n (%)				0.194				0.096
Staff	303 (16.0)	121 (12.8)	101 (13.1)		105 (26.1)	77 (31.8)	62 (26.8)	
Worker	86 (4.5)	47 (5.0)	36 (4.7)		211 (52.5)	123 (50.8)	130 (56.3)	
Farmer	381 (20.1)	216 (22.8)	167 (21.6)		64 (15.9)	22 (9.1)	26 (11.3)	
Retirees and others	1122 (59.3)	563 (59.5)	469 (60.7)		22 (5.5)	20 (8.3)	13 (5.6)	
Marital status, n (%)				0.488				0.878
Married	1529 (80.8)	769 (81.2)	640 (82.8)		375 (93.3)	225 (93.0)	213 (92.2)	
Single	363 (19.2)	178 (18.8)	133 (17.2)		27 (6.7)	17 (7.0)	18 (7.8)	

Nationality, n (%)				0.145				0.647
Han	1667 (88.1)	863 (91.1)	698 (90.3)		388 (96.5)	235 (97.1)	223 (96.5)	
Mongolian	17 (0.9)	6 (0.6)	4 (0.5)		0 (0.0)	1 (0.4)	0 (0.0)	
Zhuang	24 (1.3)	6 (0.6)	14 (1.8)		13 (3.2)	6 (2.5)	8 (3.5)	
Manchu	22 (1.2)	9 (1.0)	7 (0.9)		1 (0.2)	0 (0.0)	0 (0.0)	
Other ethnic groups	162 (8.6)	63 (6.7)	50 (6.5)		0 (0.0)	0 (0.0)	0 (0.0)	
Chronic disease history								
Liver cirrhosis, yes, n (%)	5 (0.3)	3 (0.3)	2 (0.3)	0.963	0 (0.0)	0 (0.0)	0 (0.0)	NA
Chronic hepatitis, yes, n (%)	50 (2.6)	34 (3.6)	25 (3.2)	0.351	5 (1.2)	5 (2.1)	5 (2.2)	0.611
Stroke, yes, n (%)	12 (0.6)	8 (0.8)	4 (0.5)	0.689	3 (0.7)	2 (0.8)	1 (0.4)	0.857
COPD, yes, n (%)	9 (0.5)	9 (1.0)	12 (1.6)	0.019	1 (0.2)	4 (1.7)	5 (2.2)	0.063
Myocardial infarction, yes, n (%)	7 (0.4)	3 (0.3)	1 (0.1)	0.591	3 (0.7)	4 (1.7)	0 (0.0)	0.129
Received dialysis therapy, yes, n (%)	2 (0.1)	0 (0.0)	1 (0.1)	0.576	0 (0.0)	0 (0.0)	0 (0.0)	NA
Diabetes, yes, n (%)	197 (10.4)	74 (7.8)	78 (10.1)	0.079	26 (6.5)	24 (9.9)	15 (6.5)	0.222
Hypertension, yes, n (%)	383 (20.2)	197 (20.8)	146 (18.9)	0.600	74 (18.4)	57 (23.6)	48 (20.8)	0.290
Coronary disease, yes, n (%)	69 (3.6)	41 (4.3)	36 (4.7)	0.424	8 (2.0)	15 (6.2)	8 (3.5)	0.020
Anemia, yes, n (%)	54 (2.9)	23 (2.4)	33 (4.3)	0.068	10 (2.5)	13 (5.4)	20 (8.7)	0.002
Hyperthyroidism, yes, n (%)	9 (0.5)	9 (1.0)	6 (0.8)	0.310	0 (0.0)	1 (0.4)	2 (0.9)	0.195
Hypothyroidism, yes, n (%)	7 (0.4)	4 (0.4)	1 (0.1)	0.529	0 (0.0)	1 (0.4)	0 (0.0)	0.270
Chronic pancreatitis, yes, n (%)	2 (0.1)	1 (0.1)	1 (0.1)	0.985	0 (0.0)	0 (0.0)	1 (0.4)	0.248
Osteoporosis, yes, n (%)	12 (0.6)	10 (1.1)	8 (1.0)	0.394	5 (1.2)	4 (1.7)	7 (3.0)	0.264
Ulcerative colitis, yes, n (%)	25 (1.3)	12 (1.3)	11 (1.4)	0.961	1 (0.2)	1 (0.4)	0 (0.0)	0.638
Intestinal Crohn disease, yes, n (%)	2 (0.1)	1 (0.1)	0 (0.0)	0.664	0 (0.0)	0 (0.0)	0 (0.0)	NA
Chronic biliary disease, yes, n (%)	69 (3.6)	26 (2.7)	23 (3.0)	0.389	7 (1.7)	1 (0.4)	7 (3.0)	0.090
Chronic kidney disease, yes, n (%)	4 (0.2)	3 (0.3)	2 (0.3)	0.867	0 (0.0)	0 (0.0)	0 (0.0)	NA
Tuberculosis, yes, n (%)	8 (0.4)	4 (0.4)	1 (0.1)	0.482	0 (0.0)	0 (0.0)	0 (0.0)	NA
Hospitalization information								
Family cancer history, yes, n (%)	297 (15.7)	145 (15.3)	119 (15.4)	0.958	40 (10.0)	37 (15.3)	37 (16.0)	0.043

TNM Stage, n (%)				0.937				0.001
Ⅰ	113 (6.0)	58 (6.1)	41 (5.3)		66 (16.4)	27 (11.2)	29 (12.6)	
Ⅱ	502 (26.5)	236 (24.9)	204 (26.4)		108 (26.9)	81 (33.5)	51 (22.1)	
Ⅲ	764 (40.4)	400 (42.2)	318 (41.1)		158 (39.3)	94 (38.8)	83 (35.9)	
Ⅳ	513 (27.1)	253 (26.7)	210 (27.2)		70 (17.4)	40 (16.5)	68 (29.4)	
Organ metastasis, n (%)				0.371				0.022
0	1343 (71.0)	673 (71.1)	549 (71.0)		6 (1.5)	5 (2.1)	13 (5.6)	
1	347 (18.3)	154 (16.3)	125 (16.2)		361 (89.8)	221 (91.3)	194 (84.0)	
2	97 (5.1)	59 (6.2)	43 (5.6)		31 (7.7)	12 (5.0)	18 (7.8)	
≥3	105 (5.5)	61 (6.4)	56 (7.2)		4 (1.0)	4 (1.7)	6 (2.6)	
Differentiation grade, n (%)				0.017				0.294
Well	81 (4.3)	33 (3.5)	27 (3.5)		293 (72.9)	163 (67.4)	159 (68.8)	
Moderate	1494 (79.0)	712 (75.2)	585 (75.7)		73 (18.2)	61 (25.2)	52 (22.5)	
Poor	317 (16.8)	202 (21.3)	161 (20.8)		36 (9.0)	18 (7.4)	20 (8.7)	
Radical resection, yes, n (%)	1211 (64.0)	615 (64.9)	505 (65.3)	0.774	188 (46.8)	84 (34.7)	96 (41.6)	0.011
Palliative resection, yes, n (%)	95 (5.0)	50 (5.3)	54 (7.0)	0.123	7 (1.7)	3 (1.2)	10 (4.3)	0.049
Complication of surgery, yes, n (%)	32 (1.7)	25 (2.6)	16 (2.1)	0.237	0 (0.0)	2 (0.8)	2 (0.9)	0.181
Neoadjuvant radiotherapy, yes, n (%)	19 (1.0)	14 (1.5)	16 (2.1)	0.091	6 (1.5)	5 (2.1)	4 (1.7)	0.863
Preoperative chemoradiotherapy, yes, n (%)	22 (1.2)	14 (1.5)	19 (2.5)	0.046	7 (1.7)	1 (0.4)	2 (0.9)	0.276
Adjuvant radiotherapy, yes, n (%)	79 (4.2)	32 (3.4)	23 (3.0)	0.272	5 (1.2)	1 (0.4)	6 (2.6)	0.119
Curative radiotherapy, yes, n (%)	39 (2.1)	18 (1.9)	19 (2.5)	0.713	7 (1.7)	5 (2.1)	2 (0.9)	0.555
Concurrent chemoradiotherapy, yes, n (%)	45 (2.4)	19 (2.0)	14 (1.8)	0.613	4 (1.0)	2 (0.8)	0 (0.0)	0.328
Neoadjuvant chemotherapy, yes, n (%)	37 (2.0)	22 (2.3)	24 (3.1)	0.199	7 (1.7)	5 (2.1)	2 (0.9)	0.555
Adjuvant chemotherapy, yes, n (%)	725 (38.3)	322 (34.0)	257 (33.2)	0.014	87 (21.6)	31 (12.8)	45 (19.5)	0.019
Chemotherapy for metastasis, yes, n (%)	201 (10.6)	61 (6.4)	59 (7.6)	<0.001	3 (0.7)	4 (1.7)	0 (0.0)	0.129
Curative chemotherapy, yes, n (%)	207 (10.9)	94 (9.9)	79 (10.2)	0.675	20 (5.0)	8 (3.3)	11 (4.8)	0.589
Targeted therapy, yes, n (%)	100 (5.3)	46 (4.9)	27 (3.5)	0.144	1 (0.2)	1 (0.4)	3 (1.3)	0.224
Immune therapy, yes, n (%)	62 (3.3)	11 (1.2)	6 (0.8)	<0.001	0 (0.0)	1 (0.4)	0 (0.0)	0.270

Embolization therapy, yes, n (%)	11 (0.6)	4 (0.4)	6 (0.8)	0.631	0 (0.0)	1 (0.4)	2 (0.9)	0.195
Radio frequency ablation therapy, yes, n (%)	25 (1.3)	8 (0.8)	6 (0.8)	0.334	0 (0.0)	0 (0.0)	2 (0.9)	0.061
Cryotherapy, yes, n (%)	5 (0.3)	4 (0.4)	1 (0.1)	0.510	0 (0.0)	0 (0.0)	0 (0.0)	NA
Thermotherapy, yes, n (%)	5 (0.3)	3 (0.3)	5 (0.6)	0.316	0 (0.0)	0 (0.0)	0 (0.0)	NA
Symptomatic therapy, yes, n (%)	742 (39.2)	374 (39.5)	283 (36.6)	0.390	34 (8.5)	24 (9.9)	13 (5.6)	0.219
Maintenance chemotherapy, yes, n (%)	10 (0.5)	9 (1.0)	10 (1.3)	0.112	6 (1.5)	3 (1.2)	3 (1.3)	0.959
Length of hospital stay, days, mean±SD	12.64±10.10	14.54±12.33	13.99±11.39	<0.001	13.78 (10.73)	15.69 (10.91)	15.84 (11.20)	0.028
ICU stay, yes, n (%)	246 (13.0)	188 (19.9)	124 (16.0)	<0.001	0 (0.0)	2 (0.8)	4 (1.7)	0.038
Cost, 1000 RMB yuan, mean±SD	30.03±36.84	34.05±32.64	31.76±31.77	0.014	29.41 (23.46)	35.13 (25.20)	34.70 (27.66)	0.005
KPS score, mean±SD	88.39±10.97	84.37±14.69	81.11±17.00	<0.001	90.40 (8.87)	84.75 (14.15)	82.29 (14.49)	<0.001
Nutrition-related information								
Height, cm, mean±SD	164.76±8.08	164.00±8.11	164.21±8.40	0.044	162.17 (8.53)	160.98 (8.09)	160.57 (8.52)	0.046
Weight, kg, mean±SD	63.93±10.14	59.24±10.60	55.35±11.08	<0.001	61.28 (9.32)	57.42 (10.28)	52.34 (10.23)	<0.001
Weight one month ago, kg, mean±SD	64.07±10.33	61.73±11.57	59.10±12.47	<0.001	61.54 (9.57)	59.73 (11.03)	56.18 (11.49)	<0.001
Weight six months ago, kg, mean±SD	65.54±10.57	63.53±11.64	62.07±12.16	<0.001	62.75 (10.00)	61.19 (11.03)	59.41 (12.26)	0.001
BMI, kg/m^2, mean±SD	23.48±2.83	21.97±3.26	20.45±3.45	<0.001	23.26 (2.78)	22.10 (3.29)	20.26 (3.34)	<0.001
BMI, categorical, n (%)				<0.001				<0.001
Underweight (<18.5 kg/m^2)	9 (0.5)	145 (15.3)	246 (31.8)		1 (0.2)	40 (16.5)	76 (32.9)	
Normal (18.5-23.9, kg/m^2)	1143 (60.4)	573 (60.5)	414 (53.6)		255 (63.4)	137 (56.6)	125 (54.1)	
Overweight (24-27.9, kg/m^2)	615 (32.5)	195 (20.6)	97 (12.5)		121 (30.1)	54 (22.3)	27 (11.7)	
Obesity (≥28 kg/m^2)	125 (6.6)	34 (3.6)	16 (2.1)		25 (6.2)	11 (4.5)	3 (1.3)	
Mid-arm circumference, cm, mean±SD	27.32±3.18	25.97±3.05	24.44±3.88	<0.001	26.56 (2.92)	25.70 (3.16)	23.67 (3.54)	<0.001
Triceps skinfold thickness, mm, mean±SD	17.52±7.78	16.12±7.44	15.14±8.78	<0.001	16.65 (8.64)	17.13 (9.11)	14.59 (9.77)	0.005
Mid-arm muscle circumference, cm, mean±SD	21.82±3.33	20.91±3.03	19.69±3.85	<0.001	21.33 (3.04)	20.32 (3.08)	19.09 (3.84)	<0.001
Hand grip strength, kg, mean±SD	27.67±9.69	24.24±9.34	21.79±10.63	<0.001	32.63 (16.36)	28.16 (16.39)	21.74 (14.68)	<0.001
Hand grip strength/weight ratio, mean±SD	0.43±0.14	0.41±0.14	0.40±0.18	<0.001	0.53 (0.24)	0.48 (0.25)	0.41 (0.26)	<0.001
Calf circumference, cm, mean±SD	33.83±4.14	32.32±3.68	30.96±4.21	<0.001	33.81 (3.46)	32.60 (3.28)	30.76 (3.29)	<0.001
PGSGA score, ≥4, n (%)	638 (33.7)	896 (94.6)	740 (95.7)	<0.001	170 (42.3)	229 (94.6)	222 (96.1)	<0.001

NRS2002 score, ≥3, n (%)	169 (8.9)	539 (56.9)	496 (64.2)	<0.001	73 (18.2)	172 (71.1)	190 (82.3)	<0.001
Parenteral nutritional support, yes, n (%)	474 (25.1)	291 (30.7)	296 (38.3)	<0.001	102 (25.4)	75 (31.0)	82 (35.5)	0.023
Enteral nutritional support, yes, n (%)	558 (29.5)	336 (35.5)	323 (41.8)	<0.001	99 (24.6)	73 (30.2)	82 (35.5)	0.013
No nutritional support, yes, n (%)	1252 (66.2)	557 (58.8)	395 (51.1)	<0.001	267 (66.4)	144 (59.5)	127 (55.0)	0.013
Laboratory findings								
Total protein, g/L, mean±SD	68.65±7.60	67.18±8.38	66.23±8.54	<0.001	70.10±9.25	67.45±9.05	66.52±8.52	<0.001
Creatinine, mmol/L, mean±SD	70.27±29.53	69.35±32.49	72.57±65.81	0.245	77.39±21.77	81.50±32.45	82.34±54.89	0.177
Albumin, g/L, mean±SD	40.40±13.59	38.42±5.28	37.20±6.16	<0.001	42.82±26.50	38.88±6.09	37.29±7.07	0.001
Urea nitrogen, mmol/L, mean±SD	7.42±27.55	5.99±14.30	6.88±19.05	0.298	5.89±11.90	7.35±25.14	8.03±29.23	0.446
Prealbumin, mg/L, mean±SD	224.36±75.98	205.04±87.04	187.76±81.67	<0.001	218.41±82.02	196.79±80.58	173.89±79.59	<0.001
Total bilirubin, μmol/L, mean±SD	13.76±14.14	14.87±21.22	14.24±13.79	0.222	13.31±12.13	12.92±11.24	13.80±21.03	0.812
Direct bilirubin, μmol/L, mean±SD	4.01±7.58	5.10±12.91	5.21±10.52	0.003	4.64±7.54	4.35±5.19	6.12±16.55	0.125
Transferrin, g/L, mean±SD	6.57±31.69	6.60±31.79	6.21±28.86	0.958	9.23±39.27	3.80±12.96	6.17±29.19	0.094
C-reactive protein, mg/L, mean±SD	15.16±29.14	19.96±35.11	28.00±47.25	<0.001	14.43±27.54	21.46±37.36	27.17±45.69	<0.001
Cholesterol, mmol/L, mean±SD	4.96±3.58	4.73±1.81	4.67±1.88	0.026	5.03±4.48	4.61±1.20	4.58±1.43	0.137
Glucose, mmol/L, mean±SD	5.71±2.55	5.73±2.37	5.77±2.56	0.862	5.76±3.62	5.51±1.55	5.59±2.00	0.492
Triglycerides, mmol/L, mean±SD	1.60±1.17	1.50±1.33	1.41±0.79	<0.001	1.51±1.21	1.41±0.62	1.45±1.01	0.462
AST, U/L, mean±SD	27.25±21.58	28.51±29.28	28.25±27.96	0.386	26.11±26.52	27.62±38.34	31.55±61.95	0.285
HDL-C, mmol/L, mean±SD	1.27±0.65	1.22±0.43	1.20±0.48	0.011	1.24±0.36	1.22±0.36	1.18±0.36	0.090
ALT, U/L, mean±SD	26.00±26.96	24.85±26.64	25.34±28.54	0.550	28.44±44.99	23.75±38.97	24.53±38.95	0.310
LDL-C, mmol/L, mean±SD	2.94±1.00	2.91±1.12	2.82±1.10	0.036	3.02±1.13	2.96±1.00	2.87±1.08	0.258
Hemoglobin, g/L, mean±SD	125.39±21.94	118.72±25.54	114.23±23.26	<0.001	130.03±24.43	122.05±26.44	115.94±24.36	<0.001
White blood cells, 10^9/L, mean±SD	6.20±3.30	6.40±3.03	6.90±3.71	<0.001	6.52±2.73	6.75±2.96	7.05±3.09	0.087
Neutrophils, 10^9/L, mean±SD	5.32±9.52	5.56±9.84	6.49±10.60	0.021	5.14±8.20	5.08±6.62	5.69±7.65	0.615
Lymphocytes, 10^9/L, mean±SD	2.09±4.11	1.85±3.13	1.92±4.00	0.235	2.15±4.57	1.64±1.01	1.82±4.54	0.244
Red blood cells, 10^{12}/L, mean±SD	4.43±3.57	4.16±0.63	4.12±1.64	0.006	4.43±0.71	4.29±0.76	4.35±3.18	0.634
Platelets, 10^9/L, mean±SD	214.87±84.34	232.58±99.35	236.10±102.23	<0.001	227.24±90.94	249.87±102.34	250.58±107.71	0.003

SD, standard deviation; COPD, chronic obstructive pulmonary disease; ICU, intensive care unit; KPS, the Karnofsky Performance Score; BMI, body mass index; PG-SGA, the Patient-Generated Subjective Global Assessment; NRS 2002, the Nutrition Risk Screening 2002; AST, aspartate aminotransferase; HDL-C, high density lipoprotein cholesterol; ALT, alanine transaminase; LDL-C, low density lipoprotein cholesterol.

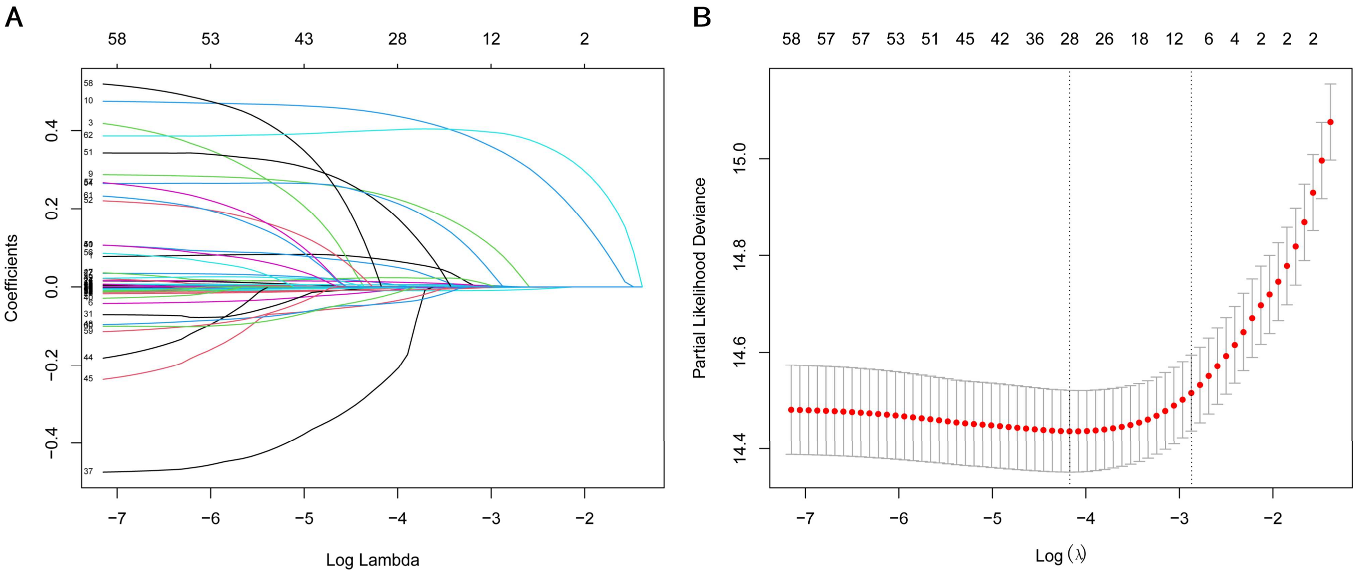

Figure S3. LASSO regression was used for dimension reduction.

Table S3. Univariate Cox Regression Analysis of the Association of the BMI, PGSGA and NRS2002 score with Overall Survival.

Characteristics	HR (95%CI)	*P*
BMI, categorical		
Underweight (<18.5 kg/m^2)	reference	-
Normal (18.5-23.9, kg/m^2)	0.67 (0.58-0.78)	<0.001
Overweight (24-27.9, kg/m^2)	0.63 (0.53-0.74)	<0.001
Obesity (≥28 kg/m^2)	0.53 (0.40-0.70)	<0.001
BMI, kg/m^2	0.96 (0.94-0.97)	<0.001
PGSGA score (≥4 vs <4)	1.36 (1.22-1.51)	<0.001
NRS2002 score (≥3 vs <3)	1.34 (1.21-1.49)	<0.001

BMI, body mass index; PG-SGA, the Patient-Generated Subjective Global Assessment; NRS 2002, the Nutrition Risk Screening 2002; HR, hazard ratio; CI, confidence interval.
